# Bayesian Variable Selection to identify QTL affecting a simulated quantitative trait

**DOI:** 10.1186/1753-6561-6-S2-S8

**Published:** 2012-05-21

**Authors:** Anouk Schurink, Luc LG Janss, Henri CM Heuven

**Affiliations:** 1 Animal Breeding and Genomics Centre, Wageningen University, PO Box 338, 6700 AH Wageningen, The Netherlands; 2 Aarhus University, DJF Department of Genetics and Biotechnology, PO Box 50, 8830 Tjele, Denmark; 3 Clinical Sciences of Companion Animals, Faculty of Veterinary Medicine, Utrecht University, PO Box 80163, 3508 TD Utrecht, The Netherlands

## Abstract

**Background:**

Recent developments in genetic technology and methodology enable accurate detection of QTL and estimation of breeding values, even in individuals without phenotypes. The QTL-MAS workshop offers the opportunity to test different methods to perform a genome-wide association study on simulated data with a QTL structure that is unknown beforehand. The simulated data contained 3,220 individuals: 20 sires and 200 dams with 3,000 offspring. All individuals were genotyped, though only 2,000 offspring were phenotyped for a quantitative trait. QTL affecting the simulated quantitative trait were identified and breeding values of individuals without phenotypes were estimated using Bayesian Variable Selection, a multi-locus SNP model in association studies.

**Results:**

Estimated heritability of the simulated quantitative trait was 0.30 (SD = 0.02). Mean posterior probability of SNP modelled having a large effect (${\hat{p}}_{i}$) was 0.0066 (95%HPDR: 0.0014-0.0132). Mean posterior probability of variance of second distribution was 0.409 (95%HPDR: 0.286-0.589). The genome-wide association analysis resulted in 14 significant and 43 putative SNP, comprising 7 significant QTL on chromosome 1, 2 and 3 and putative QTL on all chromosomes. Assigning single or multiple QTL to significant SNP was not obvious, especially for SNP in the same region that were more or less in LD. Correlation between the simulated and estimated breeding values of 1,000 offspring without phenotypes was 0.91.

**Conclusions:**

Bayesian Variable Selection using thousands of SNP was successfully applied to genome-wide association analysis of a simulated dataset with unknown QTL structure. Simulated QTL with Mendelian inheritance were accurately identified, while imprinted and epistatic QTL were only putatively detected. The correlation between simulated and estimated breeding values of offspring without phenotypes was high.

## Background

Recent developments in genetic technology enable genotyping of many individuals for thousands of markers, thereby increasing the possibility to unravel the genetic background of various economically important complex traits and disorders using genome-wide association studies. Different methods are available to perform a genome-wide association study. Bayesian Variable Selection is a powerful method in association studies [1,2], because it simultaneously estimates all SNP (Single Nucleotide Polymorphisms) effects and possible polygenic effects. Multi-locus SNP models like Bayesian Variable Selection model should therefore improve our ability to find and localize the true association between genotype and phenotype [3].

The aim of our research was to accurately identify QTL (Quantitative Trait Locus) affecting the quantitative trait and to predict breeding values of offspring without phenotypes in the simulated data of the 15^th ^QTL-MAS workshop using Bayesian Variable Selection implemented in the Bayz software [4].

## Methods

### Data

An outbred population was simulated with 1,000 generations of 1,000 individuals, which was followed by 30 generations of 150 individuals. The data used in the analysis corresponded to the last generations of the pedigree and contained 20 sire families. Each sire was mated with 10 dams and number of offspring per dam was 15 resulting in 3,000 offspring in total. Both pedigree and phenotypes of sires and dams were not provided. Genomic kinship between sires and dams indicated that sires and dams most likely descended from one population (data not shown). Only 10 out of 15 offspring per full-sib family were phenotyped for a quantitative trait that was normally distributed. All individuals were genotyped for 9,990 SNP, which were equally distributed on 5 chromosomes (size: 1 Morgan each). Monomorphic SNP (n = 2,869) and SNP with MAF (Minor Allele Frequency) <0.01 (n = 383) were excluded from the analysis. A complete description of the simulated data can be found on the website of the 15^th ^QTL-MAS workshop [5].

### QTL analysis and breeding value estimation

The model used for QTL detection and breeding value estimation simultaneously fitted polygenic and SNP effects:

$$y=\mu +Za+\Sigma_{k}X_{k}\alpha_{k}+e,$$

where **y **is the quantitative trait and *μ *is the mean; **Z **is the incidence matrix indicating for each observation the (polygenic) genetic effects by which it is influenced; **a **is the (polygenic) genetic effects with $\text{a}\sim N\left(0,\text{A}\sigma_{a}^{2}\right)$, where **A **is the numerator relationship matrix between the individuals based on pedigree and $\sigma_{a}^{2}$ is the (polygenic) genetic variance; ∑*_k_*X*_k_*α*_k _*fitted additive SNP association effects, where **α_k _**is a vector with allele substitution effects with $\sim N\left(0,\text{I}\sigma_{g_{k}}^{2}\right)$, where **I **is an identity matrix of appropriate dimensions and $\sigma_{a_{k}}^{2}$ is the additive genetic variance of SNP and **X_k _**is an incidence matrix relating allele substitution effects to observed SNP genotypes; and **e **are residuals with $\text{e}\sim N\left(0,\text{I}\sigma_{e}^{2}\right)\;{}_{{}_{}}^{}$, where **I **is an identity matrix of appropriate dimensions and $\sigma_{e}^{2}$ is the residual variance. SNP were also modelled to have a dominance effect on the simulated quantitative trait as well. However, no significant dominance effect was found (data not shown).

Bayesian Variable Selection implemented in the Bayz software [4] was used to detect QTL and predict breeding values of individuals without phenotype. The applied Bayesian Variable Selection was similar to the well-known BayesC *π *method [6], except prior of *π *had a uniform(0,1) distribution [6] while we used a slightly informative prior distribution ~ *Beta*(100,1). In Bayz [4], shrinkage of allele effects was done by applying a mixture distribution. Many SNP effects were shrunk to nearly zero to obtain high sparsity in SNP effects and only a small part of the SNP effects were less severely shrunken, thereby identifying SNP with important associations. The prior mixture distribution was:

$$\alpha_{k}\sim \{\begin{array}{c} N(0,\sigma_{g0}^{2})\;with\;probability\;\pi_{\text{0}}\\ N(0,\sigma_{g1}^{2})\;with\;probability\;\pi_{1}=(1-\pi_{0}) \end{array},$$

where the 'null' distribution modelled the majority of SNP with (virtually) no effect using prior settings *π*_0 _= 0.98 and $\sigma_{g0}^{2}=\;0.00\text{1}$. The second distribution modelled SNP with large effects where prior settings were *π*_1 _= 0.02 and $\sigma_{g1}^{2}=\;0.\text{1}$. Variances of the mixture distribution and other model effects were estimated using a uniform prior. A Bernoulli distribution specified probabilities for a SNP belonging to the 'null' or second distribution and proportions for the mixture were set to have a slightly informative prior distribution ~ *Beta*(100,1).

### Applied MCMC techniques

The model estimated a 'mixture indicator' that indicated per MCMC (Markov ChainMonte Carlo) cycle for each SNP whether it was estimated to belong to the 'null' (= 0) or second distribution (= 1). After averaging in the MCMC, a value ranging from 0 to 1 indicated the posterior probability of each SNP to have a large effect (${\hat{p}}_{i}$).

Most samplers were single site Gibbs samplers. An alternative Metropolis Hastings sampler was used to speed up mixing of estimated SNP variance components. Joint updates for 2 SNP effects and 2 'mixture indicators' were made. The Metropolis Hastings sampler updated the variance of the 'null' and second distribution thereby keeping a constant ratio (1:100) to allow for fast mixing by jointly shrinking or expanding variances together with all SNP effects. Tuning of step size from the Metropolis Hastings sampler was needed to reach an acceptance rate around 0.5.

One MCMC chain of 52,000 cycles with a burn-in period of 2,000 cycles was run, which was found sufficient to obtain accurate estimates (effective number of samples was 39.6 for polygenic genetic variance and >180 for all other model effects).

### Identification of associated SNP

Bayes Factor (BF) was used to identify associated SNP as the odds ratio between the estimated posterior and prior probabilities for a SNP:

BF=p^i(1−p^i)π1(1−π1),

where ${\hat{p}}_{i}$ = 'mixture indicator' of a SNP and *π*_1 _= prior 1/101 related to the Beta distribution. Using guidelines from Kass and Raftery [7] to judge BF, a value above 10 was considered as 'strong' evidence. SNP with BF between 3.2 and 10 were considered to be 'putative'.

In case more SNP within a region showed significant association, the size of the region and LD (Linkage Disequilibrium) (r^2^) among the SNP were used to call a single or multiple underlying QTL. When identified SNP showed clear LD blocks (r^2 ^of most SNP ≥0.7), SNP were considered to be associated with the same QTL.

## Results and discussion

**Figure 1:**
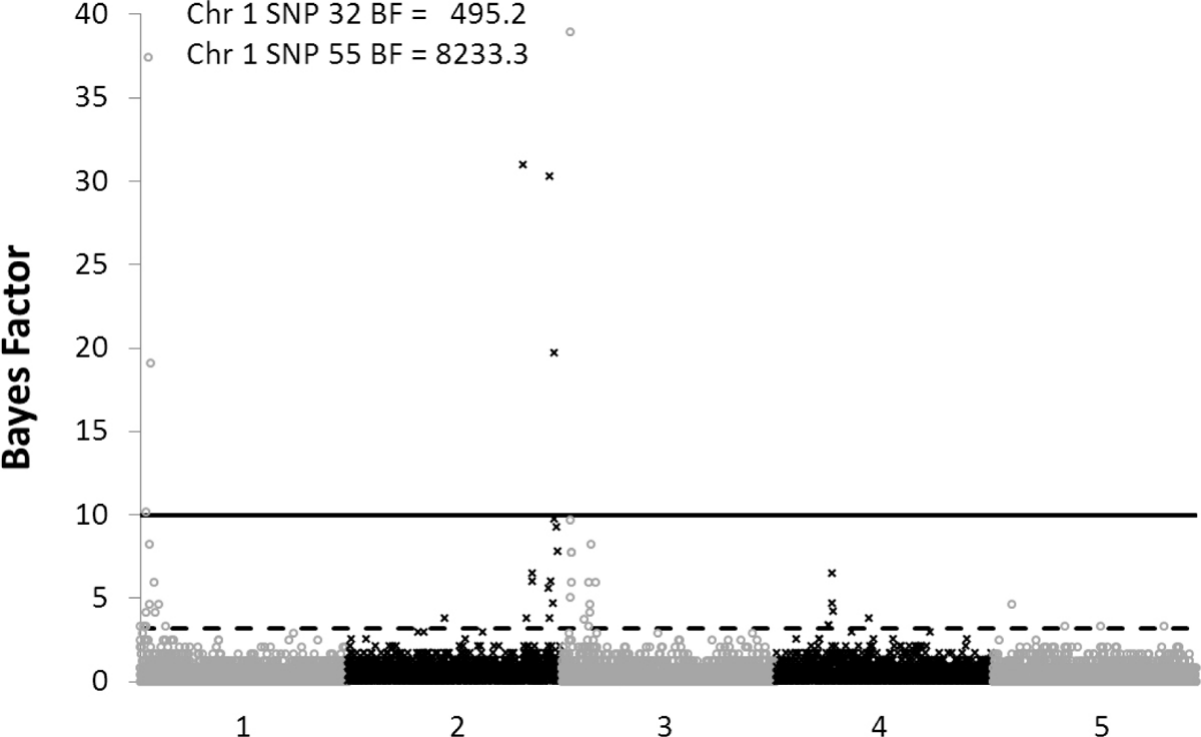
**Manhattan plot of SNP for the simulated quantitative trait**. SNP above the line are considered to be significant; SNP above the dashed line are considered to be putative.

Estimated posterior mean heritability of the simulated quantitative trait was 0.30 (SD = 0.02). The genome-wide association analysis resulted in 14 significant SNP and 43 putative SNP (Additional file 1). Taking into account the distance between SNP and their LD, we identified 7 significant QTL on chromosome 1, 2 and 3 and several putative QTL on all chromosomes. Figure 1 shows the Manhattan plot of SNP for the simulated quantitative trait. Comparison with simulated QTL presented during the QTL-MAS workshop showed that one QTL was present on chromosome 1 and two QTL on chromosome 2 and 3. Our analysis identified one of the two linked QTL in repulsion on chromosome 3, the simulated imprinted QTL on chromosome 4 and the epistatic QTL on chromosome 5 as putative. However, some putative SNP were false positive (Additional file 1). We identified QTL based on distance between SNP and their LD. However, assigning SNP as QTL based on LD patterns (SNP with low LD were considered to be separate QTL) resulted in false positive QTL. Assigning single or multiple QTL to significant SNP was not obvious, especially for SNP in the same region that were more or less in LD.

**Figure 2:**
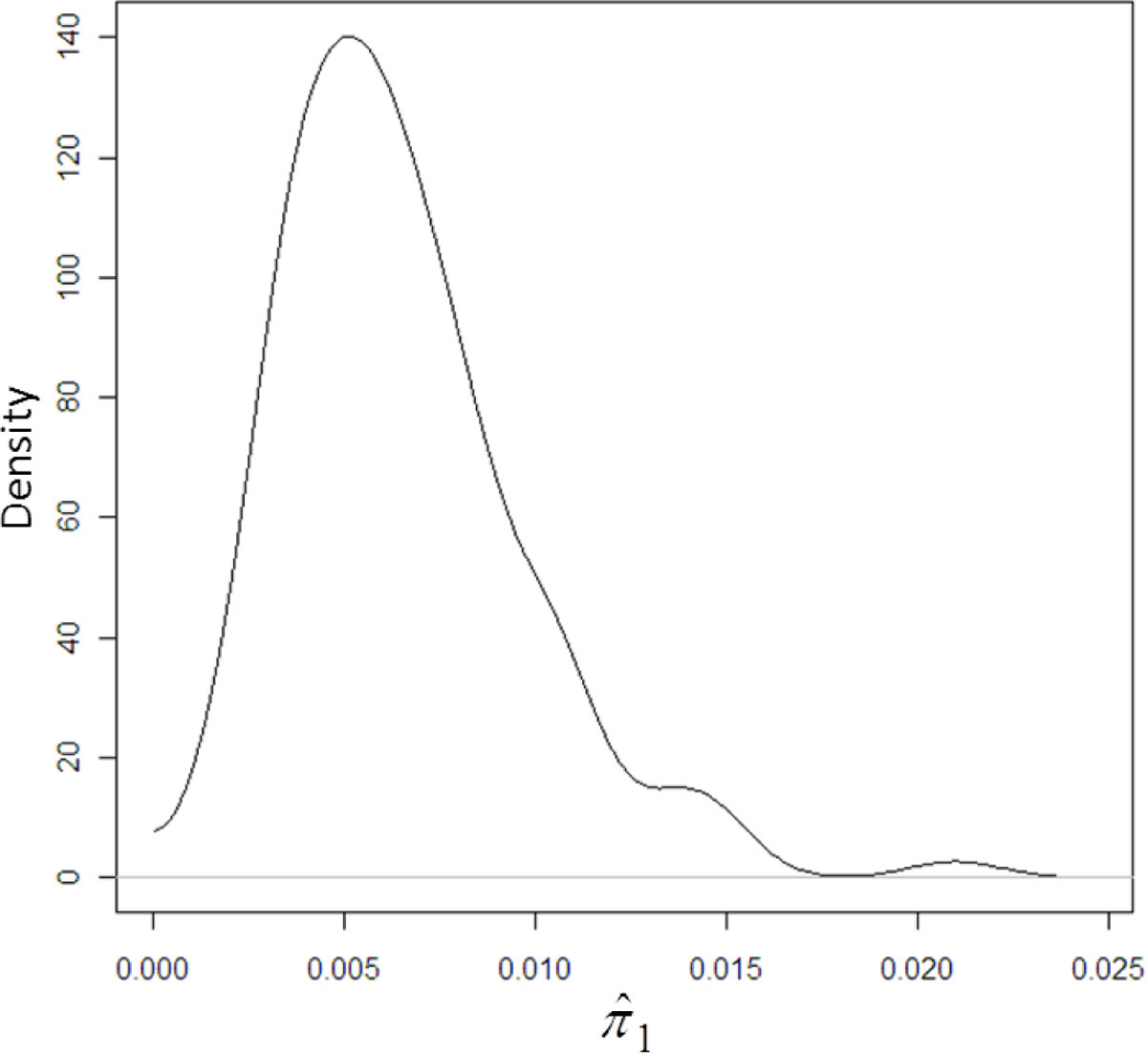
**Posterior probability density plot of proportion of SNP with large effect (**${\hat{\pi }}_{1}$**)**.

Mean posterior probability of SNP modelled having a large effect (${\hat{\pi }}_{1}$) was 0.0066 (95%HPD (Highest Probability Density) region: 0.0014-0.0132). Posterior density plot of ${\hat{\pi }}_{1}$ is given in Figure 2.

Correlation between the simulated and estimated breeding values of 1,000 offspring without phenotypes was 0.91. Comparison of all applied methods by all researchers showed that the correlation between the simulated and estimated breeding values were highest (0.85-0.94) when the data were analysed using Bayesian Variable Selection methods.

## Conclusions

Bayesian Variable Selection model showed to be a successful method for genome-wide association using dense marker maps as it identified the simulated QTL with Mendelian inheritance. Imprinted and epistatic QTL were only putatively detected. The correlation between simulated and estimated breeding values of offspring without phenotypes was high.

## List of abbreviations used

SNP: Single Nucleotide Polymorphisms; QTL: Quantitative Trait Locus; MAF: Minor Allele Frequency; LD: Linkage Disequilibrium; MCMC: Markov Chain Monte Carlo; BF: Bayes Factor; HPD: Highest Probability Density.

## Competing interests

The authors declare that they have no competing interests.

## Authors' contributions

AS analysed the data and wrote the manuscript. LLGJ developed the software (Bayz) and participated in the analyses design. HCMH conceived the project, participated in the analyses and helped to draft the manuscript.

## Supplementary Material

Additional file 1 **Overview of associated SNP**. Click here for file

## References

[B1] Hoggart CJ Whittaker JC De Iorio M Balding DJ Simultaneous analysis of all SNPs in genome-wide and re-sequencing association studies PLoS Genetics 2008 4 e1000130 10.1371/journal.pgen.1000130 18654633 PMC2464715

[B2] Sahana G Guldbrandtsen B Janss L Lund MS Comparison of association mapping methods in a complex pedigreed population Genetic Epidemiology 2010 34 455 462 10.1002/gepi.20499 20568276

[B3] Fridley BL Bayesian variable and model selection methods for genetic association studies Genetic Epidemiology 2009 33 27 37 10.1002/gepi.20353 18618760

[B4] Janss LLG Bayz manual version 2.03 Janss Biostatistics, Leiden, The Netherlands 2010 http://www.bayz.biz

[B5] Elsen JM Tesseydre S Filangi O Le Roy P Demeure O Demeure O, Elsen JM, Filangi O, Le Roy P XV^th ^QTLMAS: simulated dataset Proceedings of the XVth QTLMAS Workshop: 19-20 May 2011; Rennes France 2012 10.1186/1753-6561-6-S2-S1 PMC3363151 22640408

[B6] Habier D Fernando RL Kizilkaya K Garrick DJ Extension of the Bayesian alphabet for genomic selection BMC Bioinformatics 2011 12 186 10.1186/1471-2105-12-186 21605355 PMC3144464

[B7] Kass RE Raftery AE Bayes Factors Journal of the American Statistical Association 1995 90 773 795 10.2307/2291091

