# Supplementary material for: Bayesian Variable Selection to identify QTL affecting a simulated quantitative trait
Source: BMC Proc. 2012 May 21;6(Suppl 2):S8. doi: 10.1186/1753-6561-6-S2-S8 (PMC3363162; doi:10.1186/1753-6561-6-S2-S8)
Supplement: Additional file 1 — Overview of associated SNP. [file 1753-6561-6-S2-S8-S1.pdf]

**Overview of associated SNP.** Significant (BF>10) and putative (3.2<BF<10) SNP associated with the simulated quantitative trait, their Bayes Factor (BF), posterior probability of SNP to have a large effect ($\hat{p}_i$), mean effect of samples ($2pq\alpha_k^2$) when SNP was assigned to have a large effect. Sign of the SNP effect is arbitrary as it depends on coding of the alleles by Bayz software [4]. Features of the simulated QTL are presented and the most likely QTL position and effect are underlined.

| Chr | SNP | BF | $\hat{p}_i$ | Effect SNP | Simulated QTL |
|---|---|---|---|---|---|
| 1 | 3 | 3.3 | 0.032 | -0.67 | |
| | 28 | 3.3 | 0.032 | -0.94 | |
| | 32 | 495.2 | 0.832 | -1.54 | |
| | 49 | 3.3 | 0.032 | 1.12 | |
| | 51 | 10.1 | 0.092 | 1.05 | |
| | 53 | 4.2 | 0.040 | -1.27 | |
| | 55 | 8233.3 | 0.988 | -1.90 | |
| | 58 | 50.6 | 0.336 | 1.92 | QTL (SNP 57) with large effect, $\alpha_k$ = 2 |
| | 59 | 68.9 | 0.408 | 1.91 | |
| | 60 | 72.4 | 0.420 | 1.74 | |
| | 71 | 37.4 | 0.272 | -1.42 | |
| | 80 | 4.6 | 0.044 | 1.21 | |
| | 84 | 8.2 | 0.076 | 1.10 | |
| | 89 | 19.0 | 0.160 | -1.09 | |
| | 128 | 5.9 | 0.056 | -1.17 | |
| | 136 | 4.2 | 0.040 | -1.08 | |
| | 169 | 4.6 | 0.044 | -1.11 | |
| | 245 | 3.3 | 0.032 | -0.64 | |
| 2 | 2893 | 3.7 | 0.036 | 0.62 | |
| | 3636 | 30.9 | 0.236 | 1.28 | 2 linked QTL (SNP 3638 and 3875) in phase |
| | 3660 | 81.2 | 0.448 | 1.34 | |
| | 3680 | 3.7 | 0.036 | -0.87 | |
| | 3732 | 5.9 | 0.056 | -0.82 | |
| | 3733 | 6.4 | 0.060 | -0.91 | |
| | 3869 | 5.4 | 0.052 | 0.84 | |
| | 3873 | 30.2 | 0.232 | -1.23 | |
| | 3875 | 3.7 | 0.036 | -0.90 | 2 linked QTL (SNP 3638 and 3875) in phase |
| | 3884 | 5.9 | 0.056 | 1.04 | |
| | 3905 | 4.6 | 0.044 | -0.78 | |
| | 3914 | 19.6 | 0.164 | 1.14 | |
| | 3919 | 9.6 | 0.088 | -0.80 | |
| | 3940 | 9.2 | 0.084 | -1.00 | |
| | 3959 | 7.8 | 0.072 | 0.94 | |
| 3 | 4091 | 9.6 | 0.088 | -1.18 | |
| | 4092 | 95.3 | 0.488 | -1.59 | |
| | 4094 | 38.9 | 0.280 | -1.42 | |
| | 4097 | 5.0 | 0.048 | -1.25 | |
| | 4098 | 5.9 | 0.056 | -1.28 | |
| | 4099 | 7.8 | 0.072 | -1.31 | 2 linked QTL (SNP 4100 and 4300) in repulsion |
| | 4106 | 7.8 | 0.072 | -0.98 | |

| | | | | | |
|---|---|---|---|---|---|
| | 4238 | 3.7 | 0.036 | -0.63 | |
| | 4272 | 3.3 | 0.032 | 0.61 | |
| | 4273 | 5.9 | 0.056 | -0.62 | |
| | 4280 | 4.6 | 0.044 | 0.99 | |
| | 4288 | 4.2 | 0.040 | 0.62 | |
| | 4292 | 8.2 | 0.076 | 0.97 | 2 linked QTL (SNP 4100 and 4300) in repulsion |
| | 4331 | 5.9 | 0.056 | 0.78 | |
| 4 | 6520 | 3.3 | 0.032 | -0.74 | |
| | 6521 | 3.3 | 0.032 | -0.82 | |
| | 6554 | 4.6 | 0.044 | 0.70 | |
| | 6560 | 6.4 | 0.060 | 0.83 | |
| | 6562 | 4.2 | 0.040 | -0.76 | Maternally imprinted QTL (SNP 6644) |
| | 6931 | 3.7 | 0.036 | -0.69 | |
| 5 | 8171 | 4.6 | 0.044 | -0.72 | |
| | 8695 | 3.3 | 0.032 | 0.82 | 2 linked epistatic QTL (SNP 8726 and 9984) |
| | 9099 | 3.3 | 0.032 | 0.54 | |
| | 9673 | 3.3 | 0.032 | -0.77 | 2 linked epistatic QTL (SNP 8726 and 9984) |
